# A frequency averaging framework for the solution of complex dynamic systems

**DOI:** 10.1098/rspa.2013.0743

**Published:** 2014-06-08

**Authors:** Christophe Lecomte

**Affiliations:** Associate Member, Southampton Statistical Sciences Research Institute, University of Southampton, Southampton, UK

**Keywords:** frequency averaging, vibro-acoustics, low-mid- and high-frequency, Gaussian filter, impulse response evaluation, variance and covariance

## Abstract

A frequency averaging framework is proposed for the solution of complex linear dynamic systems. It is remarkable that, while the mid-frequency region is usually very challenging, a smooth transition from low- through mid- and high-frequency ranges is possible and all ranges can now be considered in a single framework. An interpretation of the frequency averaging in the time domain is presented and it is explained that the average may be evaluated very efficiently in terms of system solutions.

## 1. Introduction

Two regimes are often distinguished when solving structural dynamics and vibroacoustics problems in the frequency domain. Broadly speaking,

(i) the low-frequency range is a region with low modal density and low response sensitivity, whereas
(ii) the high-frequency range is a region with high modal and statistical overlaps.

The reasons for this distinction are multiple, with four salient characteristics: all details of the behaviour of the systems are not required or requested from a practical engineering point of view; the cost of modelling and solving a system in all its complexity may be excessive; some parts of the acoustic or vibratory response may be so sensitive to the system parameters that their adequacy to represent the system behaviours precisely is at best tenuous at some frequencies; and, finally, a limited set of simplified features or asymptotic behaviours may actually dominate the response in some frequency ranges.

In the low-frequency range, a relatively small number of well-defined modes are usually sufficient to describe the response of the system deterministically. Furthermore, thanks to long wavelengths of its mode shapes and propagating waves, element-based models of the system can be kept coarse, and therefore at a reasonable size, while still providing accurate representation of its behaviour. Usually, the system response is therefore essentially treated in a deterministic manner at low frequency. Relatively unexpensive physical models give accurate representation of the system behaviour and a small number of modes or waves can be evaluated and used.

The same approach can usually not be used at high frequency. Dimensionally, huge models are necessary owing to small wavelengths, the number of modes can be extremely large, and treating responses deterministically does not really make sense owing to their sensitivity. In this frequency range, statistical or average methods are usually preferred. An additional motivating factor for this choice, besides the facts that a full response could be very expensive and practically random in the presence of small system disturbances or propagating numerical errors, is that the statistical properties of the response may greatly simplify.

This fact has been most notably used in statistical energy analysis (SEA) [1,2], a now common branch of structural dynamics and vibro-acoustics whose developments were sparked by Lyon and Maidanik's paper 50 years ago [3]. Under certain conditions [4], the response of structural or acoustic components can indeed be characterized by their energy density in a frequency band and the exchange of energy between these components is proportional to the difference between their energy densities. This simplification has been used successfully to evaluate the vibration and acoustics behaviour of complex systems such as ships whose response would otherwise have been mostly untractable. A library of SEA parameters, such as coupling loss factors, has been developed for a range of material components and geometries in an analogy to the library of deterministic components developed at low frequency. Progress in the understanding of SEA and trying to extend its applicability has been made [5–8] but without generally allowing it to overlap fully with the lower frequency methods.

This problematic mid-frequency gap in understanding and modelling between low- and high-frequency regions has been long known and an area of concern. While there is an increasingly strong industrial and theoretical call for techniques that can be applied in the whole frequency range, and while both the physical or modal lower frequency techniques and the higher frequency SEA methods are pushed as involuntary candidate to fill this gap, neither approach appears to be extendable enough to cover the whole mid-frequency region. An additional difficulty is that the boundaries of applicability of low- or high-frequency methods are somewhat blurred and it may thus be hard to assess whether those are adequate in particular engineering situations.

There have been extensive efforts to describe and extend the range of applicability of the respective methods such as, starting from the higher frequencies point of view, in [9–15]. Similar efforts to encroach on the mid-frequency range have been pursued, starting from the lower frequencies point of view. For example, wave methods that allow to obtain deterministic responses over large physical domains using only a relatively small number of waves have been further developed. Since the wave characteristics can be evaluated from a detailed model of only a small portion of the domain, it is possible to part with several limitations of low-frequency methods. Alternative statistical simplifications at high frequencies have also been considered such as the representation of the system dynamic matrices by random matrices. A description of some recent developments can be found in [16–18].

Significant advances have also particularly been made in the combination of deterministic and statistical features of system characteristics. Besides the techniques to include parametric uncertainty in the low-frequency range, most notable, are relatively recent hybrid techniques such as those proposed in [19–24] that specifically deal with the mid-frequency range. The argument is that in the same structure or system, different components may see a given frequency as being either in the low- or in the high-frequency ranges. The latter ‘fuzzy’, ‘complex’ or ‘reverberant’ components are coupled to the former deterministic ‘master’ deterministic components, so that displacement variables are interfaced with energy variables and vice-versa. This allows application to specific practical engineering problems that would only be treatable with great difficulty otherwise, for example in the case of car doors that can be seen as made of relatively low-frequency frames coupled to very thin, high-frequency plates.

It is the view of the author that there remains a need for general points of view in which the various methods can be assessed, compared and extended. The work presented here intends to offer such a point of view that covers the whole frequency range. The proposed approach is to consider averaging or *filtering* of the frequency responses with tunable averaging width. On the one hand, a small averaging width leads to dealing with the system deterministically, with the frequency average being asymptotically equal to the deterministic response and zero variance. On the other hand, a larger averaging width, leads to a statistical and energy treatment of the system behaviour. Since this averaging width can be freely chosen and tuned at different values along the frequency range, it is possible to use the same analysis with a smooth transition from the low- to the mid- and high-frequency ranges.

This paper is organized in the following way. In §2, the proposed averaging process is formally described in general terms. It is then shown in §3 that the frequency average, covariance and averaged square of the response are more than theoretical concepts and that they can be evaluated in practical situations. The presentation focuses especially on the case of Gaussian averages and other possible types of averaging functions are mentioned. Illustrations are then presented and discussed in §4. Particular focus then turns to the time analysis of the averaged response in §5, and the preservation of the system energy information in §6. Finally, the efficient evaluation of the average response through matrix operations, without modal analysis, is mentioned in §7 before conclusions are drawn.

The modal expressions of the averages are very general. For any system that can be expressed in the form of equation (3.2), the frequency average of its response has the form of (3.8), where the function *S*(⋅) takes the particular values of (3.11)–(3.13) in the case of a Gaussian average. Similarly the simple expressions of the variance, covariance, and the expectation of the squares or products of the responses can be found in (3.16) and (3.28), with the values of the function *Q*(⋅) being discussed in §3*c*.

## 2. Proposed framework

The approach proposed in this paper is applicable to linear systems such as

2.1 A(ω)x(ω)=f,

where the relationships between the input and output vectors, **f** and **x** are described by a dynamic stiffness matrix **A**. This matrix and the response depend on a circular frequency parameter, *ω*=2*πf* and the interest is in the response **x**(*ω*)=**A**(*ω*)^−1^**f**(*ω*), in a frequency range $\omega \in [\omega_{\min},\omega_{\max}]$. The full response at every possible frequency within the range is however usually *not* required. An engineer may indeed only be interested in one or more particular transfer functions, such as *g*(*ω*)=**c**^*T*^**x**(*ω*), for an output vector **c**, or some combination thereof. Or, most important in the context of this article, one may only have broad interest in the frequency range: one may only be interested in a few particular modes, in the value of the response in some discrete regions, or in the average of the response magnitude or energy. The analysis in the frequency domain can be transposed in the time (*t*) domain, through the Fourier transforms

2.2 $$\underline{x}(t)=\frac{1}{2\pi }\int_{-\infty }^{\infty }x(\omega )\;e^{i\omega t}\;d\omega \;\text{and inversely},\;x(\omega )=\int_{-\infty }^{\infty }\underline{x}(t)\;e^{-i\omega t}\;dt.$$

### (a) Frequency average, averaged square and variance as computational goals

Instead of considering either the deterministic solution or the energy statistics of the system described by equation (2.1) as is typically done at low- or high-frequency ranges, it is proposed here to directly consider the *statistics* or *average* of the response **x**(*ω*) with regards to a variation in the frequency. The problem or computational goal is thus as follows: for a frequency-dependent level of uncertainty or averaging width, *a*(*ω*), and a frequency probability density function or frequency averaging function *p*_*a*_(Δ*ω*), evaluate the average and variance, that is

2.3 $$\hat{x}(\omega )=E[x(\omega +\Delta \omega )]\;\text{and}\;\mathrm{var}[x(\omega )]=E[\mathrm{abs}(x{\left(\omega +\Delta \omega )-\hat{x}(\omega )\right)}^{2}]$$

in the frequency range $\omega ,\omega_{A},\omega_{B}\in [\omega_{\min},\omega_{\max}]$. The expectation or average *E*[.] is on the random variable Δ*ω* so that, for any function *u*,

2.4 $$E[u(\omega +\Delta \omega )]=\int_{D_{p}}u(\omega +\Delta \omega )p_{a}(\Delta \omega )\;d\Delta \omega ,$$

where $D_{p}$ denotes the support of the measure *p*_*a*_(.), generally [−∞,∞] for *p*_*a*_(.) defined on the real axis. Similarly, the covariance is considered for possibly different input vectors and different frequencies, both distinguished by subindices ._A_ and ._B_ so that **A**(*ω*_A_)**x**_A_(*ω*_A_)=**f**_A_ and **A**(*ω*_B_)**x**_B_(*ω*_B_)=**f**_B_. It is defined by

2.5 $$\mathrm{cov}[x_{A}(\omega_{A}),x_{B}(\omega_{B})]=E[(x_{A}(\omega_{A}+\Delta \omega )-{\hat{x}}_{A}(\omega_{A}))(x_{B}{\left(\omega_{B}+\Delta \omega )-{\hat{x}}_{B}(\omega_{B})\right)}^{H}],$$

where the superscript .^H^ indicates the complex transpose conjugate. The variance of a vector is thus its covariance with itself. In general, when evaluating a covariance at different frequencies, the averaging width, *a*(*ω*_A_,*ω*_B_), may depend on the two frequencies.

The frequency average and covariance can be seen as the first and the second frequency moments of the response. However, since the responses are complex functions, some second moment information on the phase would be lost if only the covariance was considered. In order to remediate this, one also targets as computational objective the frequency average of the *square* of the response, that is

2.6 $$\mathrm{squ}[x(\omega )]=E[x(\omega +\Delta \omega )x{\left(\omega +\Delta \omega \right)}^{T}],$$

where the superscript .^T^ denotes the transposed vector. An additional objective, similar to the covariance, is then also the expected value of the product of responses at different frequencies or even due to different forces. It is defined as

2.7 $$\mathrm{csq}[x_{A}(\omega_{A}),x_{B}(\omega_{B})]=E[x_{A}(\omega_{A}+\Delta \omega )x_{B}{\left(\omega_{B}+\Delta \omega \right)}^{T}],$$

where the averaging width, *a*(*ω*_A_,*ω*_B_), may again depend on two parameters.

Information on the phase is then retained thanks to the transpose—rather than the complex conjugate transpose—of ${\hat{x}}_{B}(\omega_{B})$ being also considered. The frequency *first moments* or averages of the real and imaginary parts of the response are indeed

2.8 $$E[ℜ\left(x(\omega +\Delta \omega )\right)]=ℜ\left(\hat{x}(\omega )\right)\;\mathrm{and}\;E[ℑ\left(x(\omega +\Delta \omega )\right)]=ℑ(\hat{x}(\omega )),$$

and their second moments for consistent averaging widths can be derived as

2.9 $$\begin{array}{r} E[ℜ(x_{A}(\omega_{A}+\Delta \omega ))ℜ(x_{B}{\left(\omega_{B}+\Delta \omega \right)}^{T})]=\frac{1}{2}ℜ(S+P), \end{array}$$

2.10 $$\begin{array}{r} E[ℜ(x_{A}(\omega_{A}+\Delta \omega ))ℑ(x_{B}{\left(\omega_{B}+\Delta \omega \right)}^{T})]=\frac{1}{2}ℑ(S-P), \end{array}$$

2.11 $$\begin{array}{r} E[ℑ(x_{A}(\omega_{A}+\Delta \omega ))ℜ(x_{B}{\left(\omega_{B}+\Delta \omega \right)}^{T})]=\frac{1}{2}ℑ(S+P) \end{array}$$

2.12 $$\begin{array}{r} \mathrm{and}\;\;E[ℑ(x_{A}(\omega_{A}+\Delta \omega ))ℑ(x_{B}{\left(\omega_{B}+\Delta \omega \right)}^{T})]=-\frac{1}{2}ℜ(S-P), \end{array}$$

where **S**=*csq*[**x**_A_(*ω*_A_),**x**_B_(*ω*_B_)] and **P**=*E*[**x**_A_(*ω*_A_+Δ*ω*)**x**_B_(*ω*_B_+Δ*ω*)^H^]=*cov*[**x**_A_(*ω*_A_), $x_{B}(\omega_{B})]+{\hat{x}}_{A}(\omega_{A}){\hat{x}}_{B}{\left(\omega_{B}\right)}^{H}$.

### (b) Link to deterministic response and statistical energy analysis

The main advantages of the proposed solution objective, in regards to providing a consistent framework from low- to high-frequency ranges, are (i) that it can be applied in the whole frequency range and (ii) that it covers usual deterministic and statistical energy approaches as particular or asymptotic cases. The deterministic, low-frequency, approach indeed corresponds to the asymptotic case where the averaging width tends to zero

2.13 $$x(\omega )=\lim_{a(\omega )\to 0}\hat{x}(\omega )\;\mathrm{and}\;\lim_{a(\omega )\to 0}\mathrm{var}[x(\omega )]=0,$$

while the power frequency average, can be expressed in terms of the average and variance using

2.14 $$E[x(\omega +\Delta \omega )x{\left(\omega +\Delta \omega \right)}^{H}]=\mathrm{var}[x(\omega )]+\hat{x}(\omega )\hat{x}{\left(\omega \right)}^{H}.$$

The statistical simplifications encountered at high-frequency, say in the SEA theory, can thus be examined in this context. Since the averaging width *a*(*ω*) can depend on frequency, a smooth transition can be obtained through and between regions usually categorized as low-, mid- and high-frequency ranges. Such categorization is not necessary when both the first and the second frequency moments of the response are considered, although it may be very useful to make use of accurate approximations in the respective frequency ranges.

## 3. Analytical frequency average, variance and averaged square

In order to take full advantage of the proposed framework and to be able to use it in practical situations, frequency average, variance, covariance, averaged square and averaged product of responses should ideally have analytical expressions that can be evaluated accurately, robustly and cheaply.

Such exact analytical expressions of average, variance, covariance and therefore of power average have been presented in [25] and further discussed in [26–28]. For a real Gaussian function *p*_*a*_(.)=*p*^(g)^_*a*_(.), both average and variance of any transfer function can be expressed in terms of the Faddeeva function $w(z)=e^{-z^{2}}(1+(2i/\sqrt{\pi })\int_{0}^{z}e^{t^{2}}\;dt)$ [29], with different expressions depending on the imaginary parts of the system eigenvalues. In the case of purely real eigenvalues, the corresponding modal components of the averages have to be understood in a principal value sense as noted by Gautschi [30, p.188].

It is worth stressing the important subtlety that, as a function of its eigenvalue, each modal component corresponds to different analytic functions in the two half-spaces of positive and negative part of this eigenvalue. Since these functions are neither analytic continuation of each other nor equal to the principal value function on the real axis, it is essential to understand what is meant by an *undamped* modal component. If one means a modal component that has negligible damping, then one should use the expression corresponding to the limit of vanishingly small positive damping. On the other hand, in the case of an actual undamped modal component, there is no other alternative, without further information, than to use the principal value expression. Physically, if the average is evaluated through Monte Carlo sampling, in this case, there will always be samples drawn close enough to the resonance for the estimated average to *not* converge. However, if these rogue samples that are within a radius, say *ρ*, of the resonance are excluded when estimating the average, there *will* be convergence and the converged value will tend to the predicted principal value for vanishing values of *ρ*.

It was further demonstrated that all expressions could be evaluated efficiently and robustly in the whole frequency range. The theory was also extended to the case of complex normal variables [27]. In this paper, particular focus is put on the real Gaussian distribution, denoted by a superscript .^(g)^, for real argument Δ*ω*, zero mean and standard deviation *a*(*ω*) or *a*(*ω*_A_,*ω*_B_), so that one has

3.1 $$p_{a}(\Delta \omega )=p_{a}^{\left(g\right)}(\Delta \omega )=\sqrt{\frac{1}{2\pi a{\left(\omega \right)}^{2}}}\;e^{-\Delta \omega^{2}/2a{\left(\omega \right)}^{2}}.$$

Other options of averaging functions that readily fit in the proposed framework are mentioned in §3*e*. The presentation uses a modal point of view in order to retrieve the exact scalar integrals that were presented in [25,27]. The resulting average expressions may however be expressed in matrix form without requiring any explicit evaluation of eigenvalues or eigenvectors. This has been notably demonstrated in [31] where it was further shown that Krylov methods can be used to evaluate the average in a frequency range extremely efficiently. The existence of such efficient matrix evaluations is essential for practical application of the theory on dimensionally large discretized systems.

### (a) Frequency modal expression of the response

For facility of presentation, it is assumed that equation (2.1) can be expressed in an equivalent form where the dynamic stiffness matrix is a linear function of the frequency parameter, that is

3.2 $$(A_{0}^{\left(l\right)}-\omega A_{1}^{\left(l\right)})x^{\left(l\right)}(\omega )=f^{\left(l\right)}$$

with constant $A_{0}^{\left(l\right)}$ and $A_{1}^{\left(l\right)}$. This is always the case if, say, the original dynamic stiffness matrix, **A**(*ω*), is a polynomial function of *ω*. For example, in the case of a damped structural system, this matrix may be a quadratic function of *ω* as **A**(*ω*)=(**K**+*iω***C**−*ω*^2^**M**) with constant stiffness, damping and mass matrices, **K**, **C** and **M**. The matrices, output and input vectors of the equivalent form (3.2) could then be

3.3 $$A_{0}^{\left(l\right)}=\left[\begin{array}{cc} K & C\\ 0 & I \end{array}\right],\;A_{1}^{\left(l\right)}=\left[\begin{array}{cc} 0 & -iM\\ iI & 0 \end{array}\right],\;x^{\left(l\right)}(\omega )=\left[\begin{array}{c} x(\omega )\\ i\omega x(\omega ) \end{array}\right]\;\mathrm{and}\;f^{\left(l\right)}=\left[\begin{array}{c} f\\ 0 \end{array}\right].$$

The eigenvalues *ω*_*j*_ and left- and right-eigenvectors, ***ψ***^(*l*)^_*j*_ and ***ϕ***^(*l*)^_*j*_≠**0**, of the system then satisfy

3.4 $${\left[\psi_{j}^{\left(l\right)}\right]}^{T}(A_{0}^{\left(l\right)}-\omega_{j}A_{1}^{\left(l\right)})=0\;\mathrm{and}\;(A_{0}^{\left(l\right)}-\omega_{j}A_{1}^{\left(l\right)})\phi_{j}^{\left(l\right)}=0.$$

The matrix **A**^(*l*)^_1_ is assumed regular and if the system further has a full set of eigenvectors, ***ϕ***^(*l*)^_*j*_,*j*=1,…,*N*, a modal decomposition can be obtained by using the corresponding basis, $\Phi^{\left(l\right)}=[\phi_{1}^{\left(l\right)}\;\ldots \;\phi_{N}^{\left(l\right)}]$. Binormalizing the left- and right-eigenvectors with respect to the $A_{1}^{\left(l\right)}$ matrix, so that ${\Psi^{\left(l\right)}}^{T}A_{1}^{\left(l\right)}\Phi^{\left(l\right)}=I$, ${\Psi^{\left(l\right)}}^{T}A_{0}^{\left(l\right)}\Phi^{\left(l\right)}=\mathrm{diag}(\omega_{j})$, and expressing **x**^(*l*)^(*ω*)=***Φ***^(*l*)^**y**(*ω*), equation (3.2) can indeed be rewritten with diagonal matrix, as (*diag*(*ω*_*j*_)−*ω***I**)**y**(*ω*)=[***Ψ***^(*l*)^]^T^**f**^(*l*)^ which results in the following modal expression of the response:

3.5 $$x(\omega )=\sum_{j=1,\ldots ,N}\frac{\phi_{j}({\psi_{j}}^{T}f)}{\left(\omega_{j}-\omega \right)}=\sum_{j=1,\ldots ,N}\frac{P_{x}^{T}\phi_{j}^{\left(l\right)}({\left[\psi_{j}^{\left(l\right)}\right]}^{T}f)}{\left(\omega_{j}-\omega \right)},$$

for eigenvalues and modes that satisfy

3.6 $${\left[\psi_{j}^{\left(l\right)}\right]}^{T}\;A(\omega_{j})=0,\;A(\omega_{j})\phi_{j}^{\left(l\right)}=0.$$

The matrix **P**_x_ denotes the projection matrix that allows to extract the response vector **x**(*ω*) from the longer vector **x**^(*l*)^(*ω*), i.e. **x**(*ω*)=**P**_x_**x**^(*l*)^(*ω*). In the case of the damped example of equation (3.3), it is equal to **P**_x_=[**I** **0**]. While the eigenvectors, ***ϕ***_*j*_=**P**_x_***ϕ***^(*l*)^_*j*_, are therefore the first half of the linearized eigenvectors ***ϕ***^(*l*)^_*j*_ in the present example, the same expression can be found—and the theory derived below can be used—in more general cases. Note also that if the force vector was frequency dependent and could be expressed in rational form of *ω*, then the present approach could still be used with expansions similar to (3.5) and an alternative meaning for the poles.

### (b) Frequency average

In modal form, the frequency average of the response is thus the function

3.7 $$\begin{array}{r} \hat{x}(\omega )=\underset{j=1,\ldots ,N}{\Sigma }\left\{\phi_{j}({\psi_{j}}^{T}f)\int_{D_{p}}\left[\frac{1}{\left(\omega_{j}-\omega -u\right)}p_{a}(u)\;du\right]\right\} \end{array}$$

3.8 $$\begin{array}{r} =\underset{j=1,\ldots ,N}{\Sigma }\{\phi_{j}({\psi_{j}}^{T}f)S(\omega ,a,\omega_{j})\}. \end{array}$$

Ideally, the scalar integrals $S(\omega ,a,\omega_{j})=\int_{D_{p}}(1/\left(\omega_{j}-\omega -u\right))p_{a}(u)\;du$ or their combination necessitate computable exact expressions or accurate approximations which are discussed next. As discussed in [28], the effect of the averaging operation on the individual eigenvalues can be combined into the form of an (exact) averaging matrix $\hat{H}(\omega )$, so that $\hat{x}(\omega )=\hat{H}(\omega )f$, where

3.9 $$\hat{H}(\omega )=\underset{j=1,\ldots ,N}{\Sigma }\{\phi_{j}{\psi_{j}}^{T}S(\omega ,a,\omega_{j})\}.$$

The average of other transfer functions such as $i\omega \hat{x}(\omega )$ that corresponds to the system's velocity can be obtained directly from the application of the theory to the system written in the form (3.4), with matrices and vectors defined as in equation (3.3). The full averaging matrix is then simply

3.10 $${\hat{H}}^{\left(l\right)}(\omega )=\underset{j=1,\ldots ,N}{\Sigma }\{\phi_{j}^{\left(l\right)}{\psi_{j}^{\left(l\right)}}^{T}S(\omega ,a,\omega_{j})\}$$

with the same functions *S*(*ω*,*a*,*ω*_*j*_) and the vector of averaged response (and, possibly, of its averaged transfer functions of time derivatives) is ${\hat{x}}^{\left(l\right)}(\omega )={\hat{H}}^{\left(l\right)}(\omega )f^{\left(l\right)}$.

*Real Gaussian case.* In the case of a real Gaussian averaging function, averaging corresponds to applying a Gaussian filter to the response or evaluating its Weierstrass transform (see for example [32,33] and the references therein).

Since *ω* is real, the exact expressions of the integrals are [25,29,34]

3.11 $$\begin{array}{rl} S^{\left(g\right)}(\omega ,a,\omega_{j}) & =\frac{-i}{a(\omega )}\sqrt{\frac{\pi }{2}}\;w\left(\frac{\omega_{j}-\omega }{\sqrt{2}a(\omega )}\right)\;\mathrm{if}\;ℑ(\omega_{j})>0 \end{array}$$

3.12 $$\begin{array}{r} =\frac{i}{a(\omega )}\sqrt{\frac{\pi }{2}}\;w^{*}\left(\frac{{\omega_{j}}^{*}-\omega }{\sqrt{2}a(\omega )}\right)\;\mathrm{if}\;ℑ(\omega_{j})<0 \end{array}$$

3.13 $$\begin{array}{r} ={\;}^{PV}\frac{-i}{a(\omega )}\sqrt{\frac{\pi }{2}}\left\{w\left(\frac{\omega_{j}-\omega }{\sqrt{2}a(\omega )}\right)-\exp\left[-\frac{{\left(\omega_{j}-\omega \right)}^{2}}{2a{\left(\omega \right)}^{2}}\right]\right\}\;\mathrm{if}\;ℑ(\omega_{j})=0, \end{array}$$

where the complex conjugate, denoted as .*, of both the Faddeeva function and the eigenvalue in the second equation are taken and where the third equation has to be understood in a principal value sense. For example, if all the eigenvalues have a positive imaginary part, expression (3.8) of the exact average becomes

3.14 $${\hat{x}}^{\left(g\right)}(\omega )=\frac{-i}{a(\omega )}\sqrt{\frac{\pi }{2}}\left\{\underset{j=1,\ldots ,N}{\Sigma }\left[\phi_{j}({\psi_{j}}^{T}f)w\left(\frac{\omega_{j}-\omega }{\sqrt{2}a(\omega )}\right)\right]\right\}.$$

Such expression can also be expressed and evaluated directly in terms of transfer functions, the average response matrix $(-i/a(\omega ))\sqrt{\pi /2}\left\{\underset{j=1,\ldots ,N}{\Sigma }[\phi_{j}({\psi_{j}}^{T})w((\omega_{j}-\omega )/(\sqrt{2}a(\omega )))]\right\}$ or the system matrices, without requiring a modal decomposition, as discussed in [31].

### (c) Frequency variance and covariance

The frequency variance or covariance of the response is also available, from equation (2.14) and the following frequency cross-power average

3.15 E[xA(ωA+Δω)xB(ωB+Δω)H]=Σk=1,…,NΣj=1,…,N12ϕjϕkH(ψjTfA)(ψkTfB)∗×∫Dp1(ωj−ωA−u)1(ωk∗−ωB−u)pa(u) du

3.16 $$\begin{array}{r} =\underset{j,k=1,\ldots ,N}{\Sigma }\{\phi_{j}\phi_{k}^{H}({\psi_{j}}^{T}f_{A}){\left({\psi_{k}}^{T}f_{B}\right)}^{*}Q(\omega_{A},\omega_{B},a,\omega_{j},\omega_{k})\}. \end{array}$$

The integrals $Q(\omega_{A},\omega_{B},a,\omega_{j},\omega_{k})=\int_{D_{p}}(1/\left(\omega_{j}-\omega_{A}-u\right))(1/\left(\omega_{k}^{*}-\omega_{B}-u\right))p(u)\;du$ in this expression have one of two forms depending if *ω*_*j*_−*ω*_A_ and ${\left(\omega_{k}-\omega_{B}\right)}^{*}=\omega_{k}^{*}-\omega_{B}$ are equal or not [25]:

– if $\omega_{j}-\omega_{A}\neq \omega_{k}^{*}-\omega_{B}$, then, by considering partial fraction decomposition,
  3.17 $$Q(\omega_{A},\omega_{B},a,\omega_{j},\omega_{k})=\frac{S(\omega_{A},a(\omega_{A},\omega_{B}),\omega_{j})-S(\omega_{B},a(\omega_{A},\omega_{B}),\omega_{k}^{*})}{\omega_{k}^{*}-\omega_{B}-\omega_{j}+\omega_{A}},$$
– otherwise, if $\omega_{j}-\omega_{A}=\omega_{k}^{*}-\omega_{B}$,
  3.18 $$Q(\omega_{A},\omega_{B},a,\omega_{j},\omega_{k}=\omega_{j}^{*}-\omega_{A}+\omega_{B})=\int_{D_{p}}\frac{1}{{\left(\omega_{j}-\omega_{A}-u\right)}^{2}}p_{a}(u)\;du$$
  and, in the real case, one has by integration by parts,
  3.19 $$Q(\omega_{A},\omega_{B},a,\omega_{j},\omega_{k}=\omega_{j}^{*}-\omega_{A}+\omega_{B})=-\int_{-\infty }^{\infty }\frac{1}{\left(\omega_{j}-\omega_{A}-u\right)}\frac{\partial p_{a}(u)}{\partial u}\;du.$$

The frequency average power, cross-power, variance and covariance are thus directly available from expressions (3.11)–(3.13) in the first case where $\omega_{j}-\omega_{A}\neq \omega_{k}^{*}-\omega_{B}$.

*Real Gaussian case.* Considering a real Gaussian and in the first case where $\omega_{j}-\omega_{A}\neq \omega_{k}^{*}-\omega_{B}$, one finds from equations (3.17) and (3.11)–(3.13) that attention must be paid to the signs of both *ω*_*j*_ and $\omega_{k}^{*}$. For example, if the system is damped and all eigenvalues *ω*_*j*_ have positive imaginary part, their complex conjugate, $\omega_{k}^{*}$ has negative imaginary part, so that

3.20 E(g)[xA(ωA+Δω)xB(ωB+Δω)H]=−ia(ωA,ωB)π2×Σk=1,…,NΣj=1,…,NϕjϕkH(ψjTfA)(ψkTfB)∗12×w((ωj−ωA)/(2a(ωA,ωB)))+w∗((ωk−ωB)/(2a(ωA,ωB)))ωk∗−ωB−ωj+ωA.

Using equations (2.14) and (3.14), the covariance is then found to be

3.21 cov(g)[xA(ωA),xB(ωB)]=−ia(ωA,ωB)π2Σk=1,…,NΣj=1,…,NϕjϕkH(ψjTfA)(ψkTfB)∗12×w((ωj−ωA)/(2a(ωA,ωB)))+w∗((ωk−ωB)/(2a(ωA,ωB)))ωk∗−ωB−ωj+ωA−ia(ωA,ωB)π2wωj−ωA2a(ωA,ωB)w∗ωk−ωB2a(ωA,ωB)w((ωj−ωA)/2a(ωA,ωB))+w∗((ωk−ωB)/2a(ωA,ωB))ωk∗−ωB−ωj+ωAΣj=1,…,N.

Note that in the same example case of *ω*_*j*_ with positive imaginary part, if the further condition *ω*_*j*_−*ω*_A_=*ω*_*k*_−*ω*_B_ holds, then the double pole integrals equal

3.22 $$Q^{\left(g\right)}(\omega_{A},\omega_{B},a,\omega_{j},\omega_{k}=\omega_{j}-\omega_{A}+\omega_{B})=\frac{1}{aℑ\left(\omega_{j}-\omega_{A}\right)}\sqrt{\frac{\pi }{2}}K\left(\frac{ℜ\left(\omega_{j}-\omega_{A}\right)}{\sqrt{2}a},\frac{ℑ\left(\omega_{j}-\omega_{A}\right)}{\sqrt{2}a}\right)$$

where *K*(.,.) denotes the Voigt function [35], defined as the real part of the Faddeeva function $K(x,y)=ℜ[w(x+iy)]=(y/\pi )\int_{-\infty }^{\infty }\exp(-t^{2})/({\left(x-t\right)}^{2}+y^{2})\;dt$ for real *x* and *y*.

Alternatively, when $\omega_{j}-\omega_{A}=\omega_{k}^{*}-\omega_{B}$, the integral $Q^{\left(g\right)}(\omega_{A},\omega_{B},a,\omega_{j},\omega_{k}=\omega_{j}^{*}-\omega_{A}+\omega_{B})$ of equation (3.17) has a simple expression since $\partial p_{a}^{\left(g\right)}(u)/\partial u=-(u/a{\left(\omega_{A},\omega_{B}\right)}^{2})p_{a}^{\left(g\right)}(u)$. This leads indeed to the following:

3.23 $$\begin{array}{r} Q^{\left(g\right)}(\omega ,a,\omega_{j},\omega_{k}=\omega_{j}^{*}-\omega_{A}+\omega_{B})=-\frac{1}{a^{2}}\int_{-\infty }^{\infty }\frac{-u}{\left(\omega_{j}-\omega_{A}-u\right)}p_{a}^{\left(g\right)}(u)\;du \end{array}$$

3.24 $$\begin{array}{r} =-\frac{1}{a^{2}}\int_{-\infty }^{\infty }\left(1-\frac{\omega_{j}-\omega_{A}}{\left(\omega_{j}-\omega_{A}-u\right)}\right)p_{a}^{\left(g\right)}(u)\;du \end{array}$$

3.25 $$\begin{array}{r} =-\frac{1}{a^{2}}+\frac{\omega_{j}-\omega_{A}}{a^{2}}S^{\left(g\right)}(\omega_{A},a,\omega_{j}) \end{array}$$

and expressions (3.11)–(3.13) can again be used, depending on the sign of ℑ(*ω*_*j*_).

### (d) Frequency averaged square and averaged product of responses

The frequency square or averaged product of the response vectors are similarly available from

3.26 csq[xA(ωA),xB(ωB)]=Σk=1,…,NΣj=1,…,N12ϕjϕkT(ψjTfA)(ψkTfB)1(ωj−ωA−u)1(ωk−ωB−u)pa(u) du×∫Dp1(ωj−ωA−u)1(ωk−ωB−u)pa(u) du

3.27 $$\begin{array}{r} =\underset{j,k=1,\ldots ,N}{\Sigma }\{\phi_{j}\phi_{k}^{T}({\psi_{j}}^{T}f_{A})({\psi_{k}}^{T}f_{B})R(\omega_{A},\omega_{B},a,\omega_{j},\omega_{k})\}. \end{array}$$

The integrals $R(\omega_{A},\omega_{B},a,\omega_{j},\omega_{k})=\int_{D_{p}}(1/\left(\omega_{j}-\omega_{A}-u\right))(1/\left(\omega_{k}-\omega_{B}-u\right))p_{a}(u)\;du$ are thus simply $R(\omega_{A},\omega_{B},a,\omega_{j},\omega_{k})=Q(\omega_{A},\omega_{B},a,\omega_{j},\omega_{k}^{*})$ and, following the explanation of the previous section, therefore also have one of two forms depending if *ω*_*j*_−*ω*_A_ and *ω*_*k*_−*ω*_B_ are equal or not.

*Real Gaussian case.* Considering again the real Gaussian case and the particular example where the system is damped and all eigenvalues *ω*_*j*_ have positive imaginary part, the first case to consider is *ω*_*j*_−*ω*_A_≠*ω*_*k*_−*ω*_B_ when

3.28 csq(g)[xA(ωA),xB(ωB)]=−ia(ωA,ωB)π2×Σk=1,…,NΣj=1,…,NϕjϕkT(ψjTfA)(ψkTfB)12×w((ωj−ωA)/2a(ωA,ωB))−w((ωk−ωB)/2a(ωA,ωB))ωk−ωB−ωj+ωAΣj=1,…,N.

In the general real Gaussian case, one has, when *ω*_*j*_−*ω*_A_=*ω*_*k*_−*ω*_B_ and for real *ω*_A_ and *ω*_B_, that $R^{\left(g\right)}(\omega ,a,\omega_{j},\omega_{k}=\omega_{j}-\omega_{A}+\omega_{B})=Q^{\left(g\right)}(\omega ,a,\omega_{j},\omega_{k}^{*}=\omega_{j}^{*}-\omega_{A}+\omega_{B})$, whose expression is provided in equation (3.25).

### (e) Other distributions

The general expressions presented in this paper can be directly applied to a wide variety of *averaging*, *filter* or *distribution* functions. Many choices of such functions can be found in various fields such as in the theory of filters [36] or in statistics [37]. One of the most notable among those might be the uniform distribution since it has been extensively used in acoustics and vibration to model the energetic response in frequency bands or octaves. Some explicit expressions of the necessary integrals can be adapted from the expressions of the frequency average of the energy given in [38], appendix for the particular case of a proportionally damped system, i.e. when the complex poles come in particular pair combinations. Regarding the first frequency moment, i.e. the frequency average, an advantage of distributions or averaging functions that are in rational form, such as the Cauchy, Fisher's *F*, Student's *t* distributions, or the Bessel, Butterworth, Chebyshev filters, is that they may offer the computational advantage that the average can easily be expressed in matrix form, as a function of system solutions only. This can for example be used, together with the fact that the averaged input energy is proportional to the average transfer function, to approximate the averaged energy in a frequency band as in [39].

A more general approach for the average, proposed in [31] and inspired by Weideman's work on scalar rational approximations of the complex error function [40], allows working with rational matrix operations and by-passing any modal analysis, even for general non-rational distributions. Further using properties of Krylov subspaces, the main overall cost to evaluate the averages over a range a frequencies is *only a few solutions*, as demonstrated in [31] and further discussed in §8. A significant advantage worth noting is that the average at a particular frequency can be obtained with solutions at *a single shifted frequency* which corresponds to working with a system with additional damping. Any available factorization of the system dynamic stiffness matrix can thus be re-used.

The choice of which frequency averaging function to use is dependent on a user's preference and on the objectives of the application of interest. The influence of a particular choice can further be quantified, notably by examining the resulting frequency moments in the time domain. This is discussed in §§5*a*, 6 and 7. It is seen that for a constant averaging width, the averaging process results in the scaling of the exact impulse responses by the inverse Fourier transform of the averaging function. In statistics, this Fourier transform of a distribution function is called its *characteristic function* and is therefore readily available for standard distribution functions.

Complex averaging windows are also generally supported. For example, the integrals necessary for a complex Gaussian were presented in [27]. Some care must be taken since the covariance may exhibit local singularities.

## 4. Illustration of the evaluation of the frequency averages

The use of the general notions of frequency average and frequency variance can be geared towards specific applications, depending on the computational goals pursued. Various uses are illustrated in the next sections. In the current section, a benchmark problem that exhibits a mix of low- to higher frequency characteristics is first introduced. The average, variance and averaged square are then evaluated and discussed, in comparison to the system full solution.

### (a) Description of the benchmark problem

**Figure 1.**
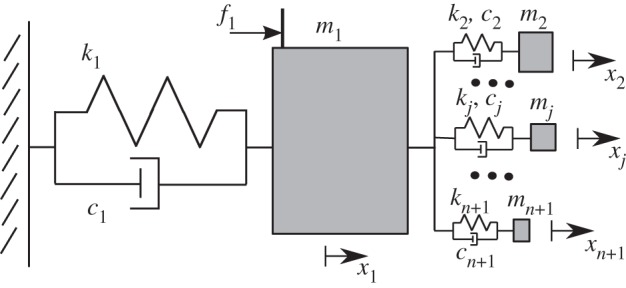
Illustration of the complex spring–mass system benchmark.

The benchmark system considered here is made of a large oscillator connected to a set of substructures, as illustrated in figure 1. Similar prototypes of complicated systems made of a large mass with many attached spring–mass systems have recurrently appeared in the literature of the last 20 years. One of the first occurrences was, for example, the undamped system that Weaver [41] used to illustrate the (ensemble) averaging approach he proposed as an alternative and extension to previous work on fuzzy and complex structures as by Soize [20] and Pierce *et al.* [21]. Although *apparently* simple, such complex systems are representative of engineering systems that are conceptually difficult to model because they exhibit a combination of low-, mid- and high-frequency characteristics. Their behaviour having actually proved to be far from simple and rather particularly interesting, they have been used in a form or another either to illustrate various theories or to study them in their own right. A salient example of the latter is the analysis in [19,42–44] and other publications of how the undamped additional spring–masses provide apparent equivalent damping to the larger mass.

Similar line of thought and motivation as that of others has been followed to select the current benchmark. Its parameters are, in particular, inspired from the first example of [19, section 7.1]. Two aspects however somewhat distinguish the current work from previous work on this type of problem. First, somewhat unusual is that the presence of both significant or smaller values of damping are supported. Second, more generally and arguably more importantly, the analysis is exact and general, so that neither approximations, such as for example an ad hoc smoothing of the modes, nor conditions on the distribution of the modes are necessary. While the chosen benchmark has damping, the proposed exact theory can also be applied without approximation to either undamped or very slightly damped systems (doing so, the remarks made in §3 about principal values must be kept in mind). It is finally worth reminding here that the averaging in the current work is on a property (the frequency) of an actual system, rather than over an ensemble of similar structures.

The details of the model are now described and the frequency average and variance are presented for varying values of the tuning parameter, *a*(*ω*). Units notations are essentially omitted (except for the time, in seconds) and may be assumed to be any consistent unit system. The main mass, *m*_1_=2 is connected with a spring, *k*_1_=2, to a fixed point and *n*=150 spring–mass pairs, *k*_*j*_, *m*_*j*_, *j*=2,…,*n*+1, are attached to it. As in [19], the individual natural frequencies, *ω*_*j*_, of the smaller attached systems are chosen randomly and uniformally within a range $\left[0,\Omega_{\max}=5\right]$. The individual masses are randomly drawn from an exponential distribution with expected total additional mass, *M*_T_. Therefore, they have probability density, $p(m_{j}n/M_{T})=\exp(-(m_{j}n/M_{T}))$ for all *j*=2,…,*n*+1 and the individual stiffness coefficients are $k_{j}=m_{j}\omega_{j}^{2}$. The actual values of the masses and springs, *m*_*j*_ and *k*_*j*_ can be found on the *j*-th line of the respective Electronic Supplementary Material Files ESM_mn and ESM_omegan, for *j*=1,…,150. Further viscous damping, *c*_*j*_=*ηk*_*j*_, is added to each spring, *j*=1,2,…,*n*+1 so that the viscous damping matrix is **C**=*η***K** for some damping factor *η*=5×10^−4^. The chosen dimension of the model is such that the ensemble of additional masses is not in any asymptotic form of complexity in the sense that the *n*=150 additional masses cannot really be considered as a good approximation of the case of a smooth modal density, notwithstanding the fact that one resonance is isolated. While this asymptotic form of an higher complexity model would not pose particular problem to the proposed framework, it is not required either.

**Figure 2.**
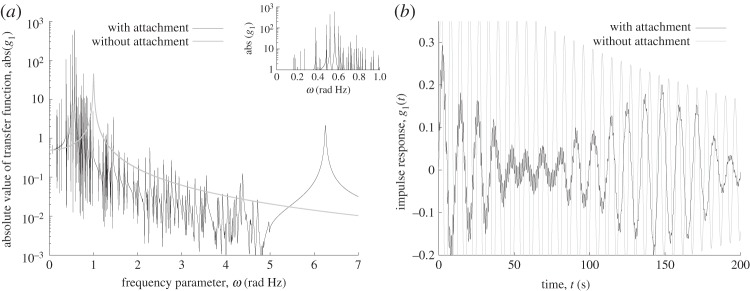
Nominal response of the main mass to a unit force in the (*a*) frequency and (*b*) time domains, with and without the additional attachments.

The interest is in the transfer function, $g_{1}(\omega )=x_{1}(\omega )=e_{1}^{T}x(\omega )$, of the main mass, *m*_1_, owing to a unit force, **f**=**e**_1_=[1 0 … 0]^T^, as well as in the impulse time response, ${\underline{g}}_{1}(t)={\underline{x}}_{1}(t)$, of the same mass. The evaluated corresponding nominal, ‘non-averaged’, functions are presented in figure 2. Some of the difficulties arising when the complexity of the system increases are illustrated on this benchmark problem and now highlighted. First, it is clear that the effect of the additional masses cannot be neglected or simply integrated in the properties of the main system. In the frequency domain, two main parts of the response are identifiable: a single well-isolated resonance in the 6≤*ω*≤7 range and an apparent blurred resonance pattern made of a combination of several resonances within the range 0≤*ω*≤5. While the latter roughly looks like a main individual resonance perturbed by a multitude of minor disturbances, such simplified vision is not really the case, since it is really the combination of all individual resonances that creates the blurred pattern. It is also not clear, de visu and qualitatively, if just one or several equivalent blurred resonances are hidden in the pattern. The situation is similarly not that simple in the time domain: the impulse response of the full system exhibits some kind of beating phenomena where its general magnitude appears to first decrease, i.e. be damped, up to about *t*=50[*s*] to only increase back up to *t*=150[*s*]. Similarly, the response at lower times exhibits a high-frequency, lower magnitude oscillation, that is superposed to a signal with wavelength equal to about 10[*s*]. Nevertheless, although the impulse response is somewhat complicated, it appears to have some hidden simplified pattern, made of only a small number of components. This situation exhibits several typical characteristics of the problems one is exposed to when working through various frequency ranges. The averaging framework proposed here offers an approach to extract such important components. Before discussing this in more detail, the use of two alternative existing approaches is presented.

**Figure 3.**
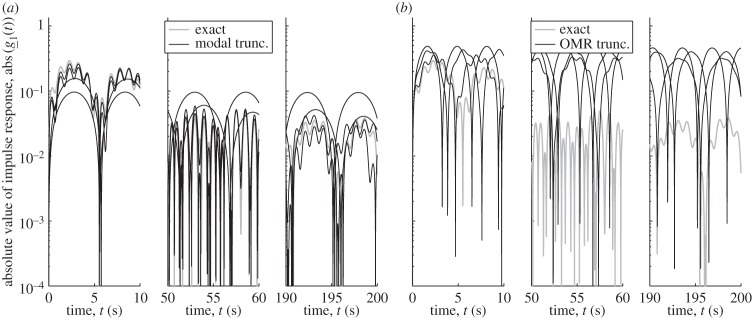
Comparison of the magnitude of the nominal (exact) response, ${\underline{g}}_{1}(t)$, to its approximation by modal truncation when (*a*) keeping 1, 2, 5 or 10 modal pairs with the highest modal density magnitude, and (*b*) keeping 1, 10 and 50 *most important* modal pairs—as defined in [45]—of the attached masses.

**Figure 4.**
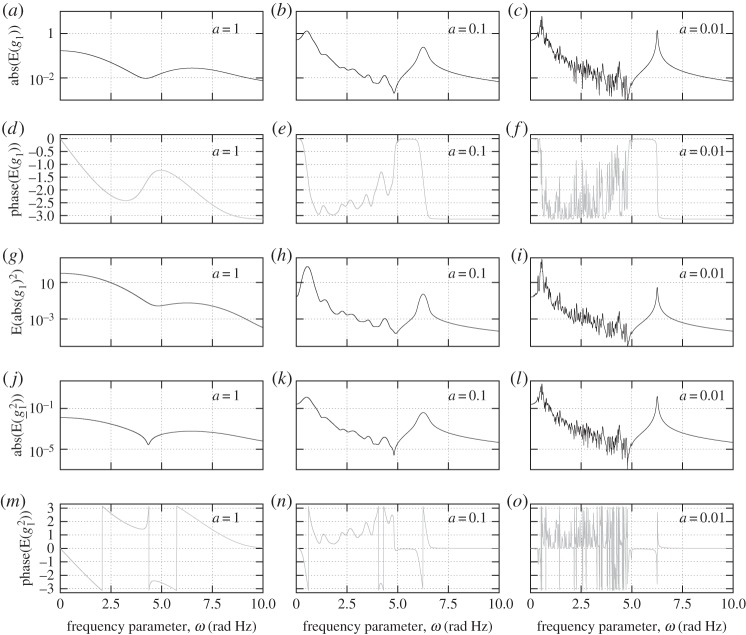
Average, $E[g_{1}(\omega +\Delta \omega )]=c^{T}\hat{x}(\omega )$, averaged absolute square, *E*[*abs*(*g*_1_(*ω*+Δ*ω*))^2^], and averaged square, *E*[*g*_1_(*ω*+Δ*ω*)^2^], of the transfer function *g*_1_(*ω*), for three values of the averaging width, *a*=1, 0.1 and 0.01.

**Figure 5.**
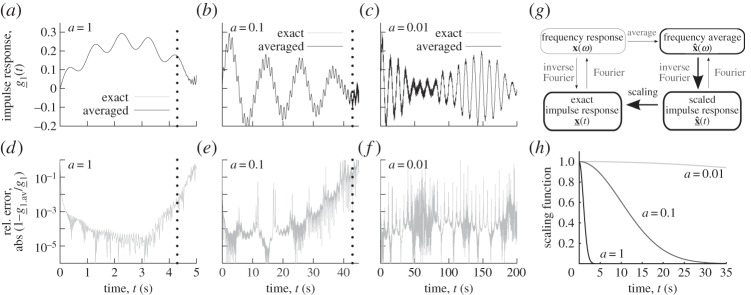
Comparison of the exact impulse response with its *‘Averaged’* estimation through frequency average, inverse Fourier transform and scaling as described in (*g*). Three averaging width, *a*=1, 0.1 and 0.01 rad Hz, are considered in plots (*a*), (*b*) and (*c*), respectively, and the corresponding relative errors are presented in plots (*d*), (*e*) and (*f*). The inverse Fourier transforms of the discretized transfer functions were evaluated through an explicit trapezoidal integral. The real Gaussian scaling functions affecting the impulse response when the inverse of the frequency average is taken are presented in (*h*).

**Figure 6.**
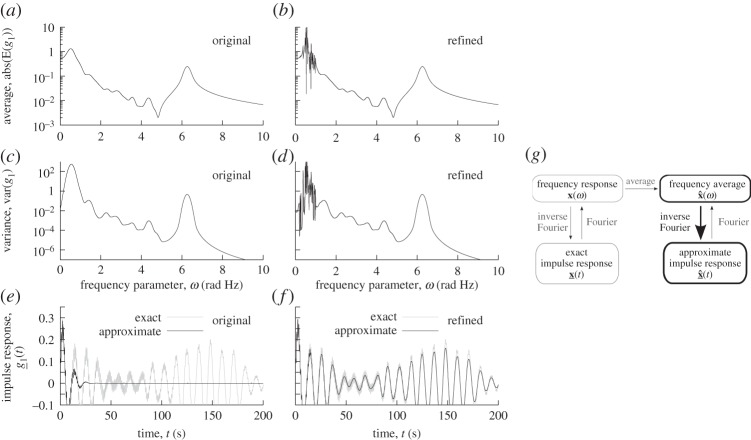
Comparison of the average, variance and impulse response functions for a constant, *original*, averaging width *a*=0.1 rad Hz and an averaging width that has been *refined* in the interval (0.1,1.1) rad Hz. The *approximate* and *exact* impulse responses are, respectively, the inverse Fourier transforms of the exact transfer function and that of its average. Both were evaluated through an explicit trapezoidal integral as for the results of figure 5.

*Alternative approaches.* An apparently evident alternative strategy to model the response of the system would be to consider the modal components of the system that *contribute most* to the response. Looking at the modal magnitudes $\mathrm{abs}(e_{1}^{T}\phi_{j}{\psi_{j}}^{T}e_{1})$ of $e_{1}^{T}x$ found from modal expression (3.5), as such a criteria, one can truncate the modal representations at a number of pairs of conjugate modes (since the response in time is real, each *ω*_*j*_ comes in pair with an eigenvalue with opposite real part, −ℜ(*ω*_*j*_)+*i*ℑ(*ω*_*j*_)). The magnitude of the impulse responses are presented in figure 3*a* for an increasing number of 1, 2, 5, 10 modal pairs whose reference eigenvalues, sorted by decreasing modal magnitude are *ω*_*j*_={0.5634+*i*0.000079, 0.5173+*i*0.000067, 6.2552+*i*0.009782, 0.6022+*i*0.000091, 0.4823+*i*0.000058, 0.7754+0.00015*i*, 0.6191+0.000096*i*, 0.3755+0.000035*i*, 0.7165+0.00013*i*, 0.8323+0.000024*i*, …}. While the predictions obtained through modal truncation are roughly similar to the actual impulse response, they are not particularly precise. For example, the time and value of the first local maximum, $\left(t,{\underline{g}}_{1}(t))=(0.3800[s],0.1126\right)$, of the impulse response is estimated as (0.3350[*s*],0.0805), when 10 pairs of modes are used. This is a 13 and 40% relative error. Another issue is that it is difficult to assess the quality of these approximations if information on the other modes is not used. Other approaches have been proposed over the last one or two decades with the objective of reducing model dimensions such that the reduced model preserves information of the original system, in particular in the time domain. Among the several options that have notably been proposed and explored by Barbone *et al.* [19], it is worth noting a connection of the current work with the smooth (mass) modal density function used to model a high-density discrete spectrum. Although starting from a different point of view, the present frequency averaging framework can indeed also be seen as offering smoothed frequency functions. Further comparison between the two points of view might therefore bring additional insight. Further efforts have also been pursued to identify and extract the *most important modes* of complicated subsystems, also with a focus on the quality of the time responses of coupled systems. The impulse response predictions obtained through the OMR algorithm of [45, Box 1, page 1669]^1^ are presented in figure 3*b* for reduced models of the attachment of dimensions 1 to 50. While such approach is better targeted than other modal truncation methods to the evaluation of impulse responses, its accuracy is still relatively limited for the amount of information preserved. Note that the possibly less precise predictions compared with those obtained via the previous modal truncation method may be explained by the fact that OMR operates *without* information on the actual system to which the complicated ensemble of attachments is connected. The predictions of both sets of impulse responses of figure 3 should be compared to the results presented in figures 4–6. It is seen that an accurate estimation of the average can be achieved with only a few *equivalent modes* and that it can provide extremely accurate time domain predictions. A practical implicit approach for evaluating such *equivalent modes* is through the rational Krylov projection method proposed in [31].

### (b) Evaluation of the frequency moments as computational goals

Rather than the full, detailed response as presented in figure 2*a*, it is the three averaged functions of order 1 and 2 that are directly targeted in the proposed framework. Such computational responses of the system are presented in figure 4 for three different values of the averaging width, *a*. Varying the value of the averaging width allows to look at the response with various interest in details, as if using a magnifying glass of various strength. For the largest value, *a*=1, all irregularities of the first *blurred resonance* are smoothed and it appears from looking at the averaged response in (*a*) that, in some way, two main resonances are present in the response. At this value of the averaging or magnifying strength, *a*=1, the average itself does not allow to differentiate between a single, *clear* resonance and a *blurred* one. Such information is however provided by the second-order averages: the ratio between the square root of the averaged square of the magnitude, in (*g*), and the absolute value of the average, in (*a*), is about 60 at the first resonance, while it is only about 8.5 at the second resonance. This information is sufficient to identify the region with higher modal density, and if desired, the averaging width can be refined. Doing so reveals more details of the response as seen in (*b*) where about 10 distinct *resonances* are apparent. Pushing further the identification of details by decreasing the averaging width to *a*=0.01 leads to a much less regular response in (*c*). This lightest touch average is however notably smoother than the full detailed solution of figure 2: the minor resonances that are not important in the response have been smoothed out.

The averages thus provide qualitative information about the location and density of resonances. As such, they are useful by themselves and one can use them to refine the analysis, i.e. to decrease the averaging width, in regions of interest. The effect of averaging can also be characterized in the time domain, as now described in §5.

## 5. Time domain analysis and impulse response of the averaged responses

In this section, the interpretation and effect of the frequency averaging in the time domain is discussed. Constant and variable averaging width, *a*(*ω*), are studied in turn.

### (a) Impulse response from frequency averaging with constant width

Properties of convolutions can be used to evaluate time responses when the averaging width is constant. This is described and illustrated in the two following sections, in particular in the case of Gaussian averaging.

#### (i) Description of the approach to evaluate time responses

First, for a constant *a*(*ω*)=*a*, averaging the full response, **x**(*ω*), is equivalent to evaluating its convolution with the averaging window or probability density function *p*_*a*_(Δ*ω*). The inverse Fourier transform of the average $\hat{x}(\omega )$ is therefore equal to the product of the exact impulse response by the inverse Fourier transform of *p*_*a*_(Δ*ω*). The latter is called the *characteristic function* of the probability density function, *p*_*a*_(.), in statistics.

Specifically, considering the Fourier transform, $\underline{\hat{x}}(t)$, of the frequency average transfer function vector, $\hat{x}(\omega )$, one has in general the following filtered impulse response:

5.1 $$\underline{\hat{x}}(t)=\frac{1}{2\pi }\underset{j=1,\ldots ,N}{\Sigma }\left\{\phi_{j}({\psi_{j}}^{T}f)\int_{-\infty }^{\infty }\int_{D_{p}}\left[\frac{1}{\left(\omega_{j}-\omega -u\right)}p_{a}(u)\;du\right]e^{i\omega t}\;d\omega \right\}.$$

Since *a*(*ω*)=*a* is constant, the change of variable *ω*′=*ω*+*u* gives the product of the impulse response of the system by the characteristic function of *p*_*a*_(.), i.e.

5.2 $$\begin{array}{rl} \underline{\hat{x}}(t) & =\frac{1}{2\pi }\underset{j=1,\ldots ,N}{\Sigma }\left\{\phi_{j}({\psi_{j}}^{T}f)\int_{D_{p}}\int_{-\infty }^{\infty }\left[\frac{1}{\left(\omega_{j}-\omega^{\prime }\right)}e^{i\omega^{\prime }t}d(\omega^{\prime })p_{a}(u)\;e^{-iut}\;du\right]\right\} \end{array}$$

5.3 $$\begin{array}{r} =\underline{x}(t)\int_{D_{p}}p_{a}(u)\;e^{-iut}\;du. \end{array}$$

Equation (5.3) offers a strategy to evaluate the time response of the system, which does not require the knowledge of the details of the full system, but only its average response. The proposed strategy, valid for times at which the values of the scaling function $\int_{D_{p}}p_{a}(u)\;e^{-iut}\;du$ are large enough, is illustrated in figure 5 for the real Gaussian case. It has three main steps:

(i) evaluate the frequency average, $\hat{x}(\omega )$;
(ii) evaluate its inverse Fourier transform, $\underline{\hat{x}}(t)$ and
(iii) scale this time function, using equation (5.3) in general and (5.7) in the particular real Gaussian case, to estimate the impulse response, $\underline{x}(t)$.

Each of these operations has the option to be evaluated in various manners. For example, while the frequency average could be evaluated by using the modal decomposition used in the derivation in this paper, an efficient matrix alternative based on rational approximations and Krylov subspaces that has been proposed by Lecomte [31] allows by-passing any modal analysis and has been successfully applied by the author to large systems in this context of evaluating time responses as proposed here. Similarly, the inverse Fourier transform could be evaluated by considering individual modes of a modal decomposition or numerically by sampling the average in the frequency domain. The evaluation through modal decomposition can also be applied to the precise approximate reduced models of [31].

*Real Gaussian case.* In the particular real Gaussian case when *p*_*a*_(*u*)=*p*^(g)^_*a*_(*u*), the Fourier transform of a real Gaussian averaging function is another real Gaussian function: starting from

5.4 $$\int_{-\infty }^{\infty }p_{a}^{\left(g\right)}(u)\;e^{-iut}\;du=\int_{-\infty }^{\infty }\sqrt{\frac{1}{2\pi a^{2}}}\;e^{-u^{2}/(2a^{2})}\;e^{-iut}\;du$$

one can complete the square in the exponential argument and use Cauchy integral theorem to find

5.5 $$\begin{array}{r} \int_{-\infty }^{\infty }p_{a}^{\left(g\right)}(u)\;e^{-iut}\;du=\int_{-\infty }^{\infty }\sqrt{\frac{1}{2\pi a^{2}}}\;e^{-{\left(u/(\sqrt{2}a)+ita/\sqrt{2}\right)}^{2}}e^{-t^{2}a^{2}/2}\;du \end{array}$$

5.6 $$\begin{array}{r} =e^{-t^{2}a^{2}/2}\int_{-\infty }^{\infty }\sqrt{\frac{1}{2\pi a^{2}}}\;e^{-{\left(u/(\sqrt{2}a)\right)}^{2}}\;du=e^{-t^{2}a^{2}/2}. \end{array}$$

The Gaussian filtered impulse response is thus

5.7 $${\underline{\hat{x}}}^{\left(g\right)}(t)=\underline{x}(t)\;e^{-t^{2}a^{2}/2}.$$

This shows that Gaussian frequency averaging for constant *a*(*ω*)=*a* preserves the nominal impulse response at time zero, i.e. $\underline{\hat{x}}(0)=\underline{x}(0)$ and that the smaller the value of *a*, the better is ${\underline{\hat{x}}}^{\left(g\right)}(t)$ an approximation of $\underline{x}(t)$ at small times.

Note finally that the current discussion provides a description of the eigenfunctions of the Gaussian averaging operator. This is detailed in appendix B in the electronic supplementary material.

#### (ii) Illustration of the approach to evaluate time responses

The estimations of figure 5 were evaluated through a modal decomposition and the discretization of the frequency average in a wide but finite range of frequencies. The relative error on the estimated impulse responses is about 10^−3^ or less in most of the time ranges corresponding to each of the averaging widths. The increase in the error at the end of the two first time ranges, i.e. the intervals (0,5)[*s*] for *a*=1 and (0,45)[*s*] for *a*=0.1, is due to two reasons: first, the precision of the evaluation of the average and, mostly, of the inverse Fourier transform, and, second, the limits of the finite precision arithmetics. Both the evaluation of the average and inverse Fourier transform can be made much more precise, particularly by using the method of Lecomte [31]. The finite precision arithmetic remark corresponds to a more fundamental fact: since the *impulse response* $\underline{\hat{x}}(t)$ has to be scaled by the inverse Gaussian *e*^*t*^2^*a*^2^ /2^ to estimate $\underline{x}(t)$, this estimation will be marred by round-off effects if $\underline{\hat{x}}(t)$ is represented in finite precision. The estimations for smaller values of *t* and the scaling factor remain precise as is shown in figure 5 where the vertical dotted lines correspond to values of this factor equal to *e*^*t*^2^*a*^2^ /2^=10^4^. If an engineer, who would for example want to design a structure that is only moderately affected by a shock or turbulence, is only interested in the maximum and minimum values of the impulse response during a given time period after some impact, as in (*a*), he or she can thus define the maximum averaging width possible that provides accurate response in the smallest time range of interest. The discussion of this section provides some explanation of the Gaussian frequency averaging process in that the smoothed out details and irregularities of the transfer functions correspond to details of the response at larger times. Averaging with other distributions, as those discussed in §3*e*, leads to different but similar interpretations since the characteristic functions of these distributions—their inverse Fourier transforms—has other properties.

Nevertheless their being smoothed out, information about the details of the transfer functions does not disappear in the averaging process, at least when the second-order averages—averaged square and variance—are considered. The information about the total transient energy, including at larger times, is indeed preserved and retrievable. This will be discussed in §6. Before that, the case of variable averaging width is briefly discussed.

### (b) Time domain analysis using a variable averaging width

It was mentioned in §2*b* that the *low-frequency* focus corresponds to responses that have zero or small variance. The discussion of the previous section provides the further interpretation that their *deterministic* character can be understood as well defined—rather than blurred—transfer functions and consequently refined impulse responses at small times. The alternative *high-frequency* focus corresponds instead to *statistical*, blurred transfer functions and a more and more imprecise notion of energy at larger and larger times. It is now stressed how the focus of the analysis can be qualitatively pushed into the direction of a more deterministic character, by pinpointing frequency regions.

In general, since the averaging width, *a*(*ω*), can be frequency dependent, it can indeed be used to gear the analysis into different focus in different frequency ranges and provide a smooth transition from low- to high-frequency as shown in [31]. Another application, illustrated in figure 6, is presented here. The frequency average response is by itself an approximation of the exact response, in the sense that the averaging process may allow to get rid of unimportant features of the response. As noted in the previous sections, this notion of unimportance is related to an accurate time response of the system at small times and a small frequency variance. Steps for reducing the frequency variance may therefore lead to a focus on more important features of the system response.

The analysis presented in figure 4 for *a*=0.1 is examined again. The corresponding inverse Fourier transform of the average is presented in figure 6*e* and is seen to rapidly lose precision for increasing time. This can be compared to figure 4*b*, where the Gaussian scaling was applied. Since the frequency variance is relatively the largest in the region *ω*<1, reducing the averaging width, i.e. refining the response, in this lower frequency region may consequently achieve a generally better approximation in the time domain.

A constant refined value of *a*=0.004 is chosen in the interval 0.2≤*ω*≤1 (rad Hz) and is connected to the initial *a*=0.1 everywhere else through a linear variation at the edges *ω*∈(0.1,0.2) and (1,1.1) (rad Hz). With this choice, the average reveals a number (about 30) of resonances in the refined interval and globally reduces the variance there. As can be seen in plot ( *f*), this significantly increases the quality of the approximate response over a larger region of time and, while qualitative, this study illustrates again the link between averaging and precise response at small time. Attention now turns to the *imprecise* or energy part of the responses that are assessed by the second-order averaged square and variance.

## 6. Repartition of the energy in the system response

It is shown in this section that the total transient energy, which transits through part of a system during impulse, can be retrieved from a combination of its first and second frequency moments obtained with constant averaging width, *a*(*ω*)=*a*. Equivalence theorems of two kinds are first highlighted. Exact expressions of the energy in terms of the frequency moments are then presented and discussed. It is notably shown how the energy is partitioned in terms of the first and the second moment contributions.

### (a) Energy equivalence theorems

The evaluation of the total impulse energy through averaged functions is based on theorems of equivalence of the energy in the three domains, namely the domains of time, frequency and frequency average. The known equivalence between the time and frequency domains is reminded in the next section while the equivalence between the nominal and average frequency domains is introduced in §6*a*(ii).

#### (i) Time and frequency energy density equivalence

The first kind of equivalence between the frequency and time densities is provided by Plancherel's theorem below, whose proof is provided in appendix A (in the electronic supplementary material) for completeness.

**Theorem 6.1**

*Using the notations of equation (2.2)
*

6.1 $$X=\int_{-\infty }^{\infty }\underline{x}(t)\underline{x}{\left(t\right)}^{T}\;dt=\frac{1}{2\pi }\int_{-\infty }^{\infty }x(\omega )x{\left(\omega \right)}^{H}\;d\omega .$$

Its application to time derivatives gives the expressions needed for the evaluation of the kinetic energy, e.g. as follows.

**Corollary 6.2**

*Using the notations of equation (2.2)*

6.2 $$D=\int_{-\infty }^{\infty }\frac{\partial \underline{x}(t)}{\partial t}{\frac{\partial \underline{x}(t)}{\partial t}}^{T}\;dt=\frac{-1}{2\pi }\int_{-\infty }^{\infty }\omega^{2}x(\omega )x{\left(\omega \right)}^{H}\;d\omega .$$

#### (ii) Frequency and frequency average energy density equivalence

The second kind of equivalence concerns the frequency integrals of functions of the responses and of their frequency average. In particular, the following theorem for the products of responses is used for the evaluation of the total potential energy in §6*b*.

**Theorem 6.3**

*The integrated quadratic term defined in equation (6.1) is equal to the integrated frequency averaged quadratic term, i.e.
*

6.3 $$X=\frac{1}{2\pi }\int_{-\infty }^{\infty }x(\omega )x{\left(\omega \right)}^{H}\;d\omega =\frac{1}{2\pi }\int_{-\infty }^{\infty }E[x(\omega +\Delta \omega )x{\left(\omega +\Delta \omega \right)}^{H}]\;d\omega =\hat{X},$$

*where* E[**x**(*ω*+Δ*ω*)**x**(*ω*+Δ*ω*)^H^] *denotes the frequency average on the frequency shift* Δ*ω for any probability density function, p*_*a*_(Δ*ω*), *and constant standard deviation, a,*

6.4 $$E[x(\omega +\Delta \omega )x{\left(\omega +\Delta \omega \right)}^{H}]=\int_{D_{p}}x(\omega +\Delta \omega )x{\left(\omega +\Delta \omega \right)}^{H}p_{a}(\Delta \omega )\;d\Delta \omega .$$

**Proof.**

Substituting the expression of the frequency average, considering the constance of *a*, and changing the variable *ω*′=*ω*+Δ*ω*, one finds $\hat{X}=\int_{D_{p}}[(1/2\pi )\int_{-\infty }^{\infty }x(\omega^{\prime })x{\left(\omega^{\prime }\right)}^{H}d\omega^{\prime }]p_{a}(\Delta \omega )\;d\Delta \omega$ and one can then integrate successively with respect to *ω*′ and *d*Δ*ω* to find *X*. □

The integrals match similarly when the time derivatives of equation (6.2) are considered, i.e.

**Theorem 6.4**

*The integrated term defined in equation (6.2) is equal to its integrated frequency average, i.e.
*

6.5 $$D=\frac{-1}{2\pi }\int_{-\infty }^{\infty }\omega^{2}x(\omega )x{\left(\omega \right)}^{H}\;d\omega =\frac{-1}{2\pi }\int_{-\infty }^{\infty }E[{\left(\omega +\Delta \omega \right)}^{2}x(\omega +\Delta \omega )x{\left(\omega +\Delta \omega \right)}^{H}]\;d\omega =\hat{D}$$

*for constant averaging width a*(*ω*)=*a.*

The proof is similar to that of theorem 6.3 and this property is used for the evaluation of the total kinetic energy in §6*c*.

These and similar equivalence properties can be applied to evaluate the energy terms of a system, just from the knowledge of its frequency average first and second moments targeted in the proposed framework. It is now demonstrated how this can be done.

### (b) Repartition and retrieval of potential energy

The particular case of a typical dynamic stiffness matrix **A**(*ω*)=(**K**+*iω***C**−*ω*^2^**M**) is considered for illustrative purpose. Examining a transient response of the system, since the potential energy of the whole system at any time *t* is $U(t)=(1/2)\underline{x}{\left(t\right)}^{T}K\underline{x}(t)$, its integral over time is equal to $U=\int_{-\infty }^{\infty }U(t)\;dt=(1/4\pi )\int_{-\infty }^{\infty }x{\left(\omega \right)}^{H}Kx(\omega )\;d\omega$.

*Full system energy.* Using theorem 6.3, and denoting *k*_*kj*_ the element of **K** in *k*th row and *j*th column, one has that

6.6 $$\begin{array}{r} U=\frac{1}{4\pi }\underset{k=1,\ldots ,N}{\Sigma }\;\underset{j=1,\ldots ,N}{\Sigma }\left[k_{kj}\left(\int_{-\infty }^{\infty }x_{j}(\omega )x_{k}{\left(\omega \right)}^{*}\;d\omega \right)\right] \end{array}$$

6.7 $$\begin{array}{r} =\frac{1}{4\pi }\underset{k=1,\ldots ,N}{\Sigma }\;\underset{j=1,\ldots ,N}{\Sigma }\left[k_{kj}\left(\int_{-\infty }^{\infty }E[x_{j}(\omega +\Delta \omega )x_{k}{\left(\omega +\Delta \omega \right)}^{*}]\;d\omega \right)\right]=\hat{U}. \end{array}$$

This expression can further be expanded in terms of the average and variance of the response by using equation (2.14) which gives

6.8 $$\begin{array}{rl} U=\hat{U} & =\frac{1}{4\pi }\left\{\underset{k=1,\ldots ,N}{\Sigma }\;\underset{j=1,\ldots ,N}{\Sigma }\left[k_{kj}e_{j}^{T}\left(\int_{-\infty }^{\infty }\mathrm{var}[x(\omega )]\;d\omega \right)e_{k}\right]\right.\\ & \;+\left.\underset{k=1,\ldots ,N}{\Sigma }\;\underset{j=1,\ldots ,N}{\Sigma }\left[k_{kj}\left(\int_{-\infty }^{\infty }{\hat{x}}_{j}(\omega ){\hat{x}}_{k}{\left(\omega \right)}^{H}\;d\omega \right)\right]\right\} \end{array}$$

or, in compact matrix form,

6.9 $$U=\hat{U}=\frac{1}{4\pi }\left\{\mathrm{tr}\left[K\left(\int_{-\infty }^{\infty }\mathrm{var}[x(\omega )]\;d\omega \right)\right]+\int_{-\infty }^{\infty }\hat{x}{\left(\omega \right)}^{H}K\hat{x}(\omega )\;d\omega \right\},$$

where $\mathrm{tr}\left[.\right]$ denotes the trace of a matrix, i.e. the sum of its diagonal coefficients.

*Component energy.* The integrated potential energy of a *component* of the system can similarly be expressed in terms of frequency averages. For example, if the system is decomposed into two components, distinguished by indices ‘A’ and ‘B’, such that

6.10 $$x(\omega )=[V_{A}\;V_{B}]\left[\begin{array}{c} x_{A}(\omega )\\ x_{B}(\omega ) \end{array}\right]\;\mathrm{and}\;\left[\begin{array}{c} x_{A}(\omega )\\ x_{B}(\omega ) \end{array}\right]={\left[W_{A}\;W_{B}\right]}^{T}x(\omega ),$$

then the energy of the component *j* = A or B is equal to

6.11 $$U_{j}=\frac{1}{4\pi }\left\{\mathrm{tr}\left[K_{jj}\left(\int_{-\infty }^{\infty }\mathrm{var}[x_{j}(\omega )]\;d\omega \right)\right]+\int_{-\infty }^{\infty }{\hat{x}}_{j}{\left(\omega \right)}^{H}K_{jj}{\hat{x}}_{j}(\omega )\;d\omega \right\},$$

where $\mathrm{var}[x_{j}(\omega )]=W_{j}^{T}\mathrm{var}[x(\omega )]W_{j}^{*}$, ${\hat{x}}_{j}(\omega )=W_{j}^{T}\hat{x}(\omega )$ and $K_{jj}=V_{j}^{H}KV_{j}$. Similarly, the integrated potential energy of the coupling between the two components is equal to *U*_AB_+*U*_BA_ where

6.12 $$U_{jk}=\frac{1}{4\pi }\left\{\mathrm{tr}\left[K_{jk}\int_{-\infty }^{\infty }\mathrm{cov}[x_{k}(\omega ),x_{j}(\omega )]\;d\omega \right]+\int_{-\infty }^{\infty }{\hat{x}}_{j}{\left(\omega \right)}^{H}K_{jk}{\hat{x}}_{k}(\omega )\;d\omega \right\}$$

for $K_{jk}=V_{j}^{H}KV_{k}$ and *j*,*k*= A or B.

For example, the integrated potential energy of a component made of a single degree of freedom, say **V**_A_=**e**_*j*_ where **e**_*j*_ is the unit vector with zero components except for 1 at its *j*th component, is

6.13 $$U_{A}=\frac{k_{jj}}{4\pi }\left\{\int_{-\infty }^{\infty }(\mathrm{var}[x_{j}(\omega )]+|{\hat{x}}_{j}(\omega ){|}^{2})\;d\omega \right\}=\frac{k_{jj}}{4\pi }\int_{-\infty }^{\infty }E[|x_{j}(\omega +\Delta \omega ){|}^{2}]\;d\omega .$$

*Real Gaussian case.* In the real Gaussian case, the values of the integral function *S*^(g)^(*ω*,*a*,*ω*_*j*_) were given in equations (3.11)–(3.13) and those of *Q*^(g)^(*ω*,*a*,*ω*_*j*_,*ω*_*k*_) can be found in equations (3.17)–(3.25), with appropriate substitution of the right expression of the *S*^(g)^ function, based on the sign of the imaginary part of its last argument (i.e. the sign of ℑ(*ω*_*j*_) and $ℑ(\omega_{k}^{*})$). As discussed in §8, the average vector or scalar functions, ${\hat{x}}^{\left(l\right)}(\omega )$ and ${\hat{x}}_{j}^{\left(l\right)}(\omega )$, can also be directly evaluated without requiring any modal analysis and therefore, by bypassing any evaluation or use of *S*^(g)^(*ω*,*a*,*ω*_*j*_).

### (c) Repartition and retrieval of kinetic energy

The integrated kinetic energy can also be expressed in either time or frequency domains. The expressions of the variance and products cannot be used directly owing to the presence of the *ω*^2^ term in the right-hand side of the last equation. The integrand must first be modified. Starting from equation (3.5), one finds

6.14 $$\omega x(\omega )=\underset{j=1,\ldots ,N}{\Sigma }\phi_{j}({\psi_{j}}^{T}f)\frac{\omega }{\left(\omega_{j}-\omega \right)}=\underset{j=1,\ldots ,N}{\Sigma }\left\{\phi_{j}({\psi_{j}}^{T}f)\left[\frac{\omega_{j}}{\left(\omega_{j}-\omega \right)}-1\right]\right\},$$

which allows to evaluate the corresponding frequency average in terms of known integral functions *S*(*ω*,*a*,*ω*_*j*_) and *Q*(*ω*,*a*,*ω*_*j*_,*ω*_*k*_). Indeed, for a general pdf *p*_*a*_(Δ*ω*)

6.15 $$\begin{array}{rl} & E[{\left(\omega +\Delta \omega \right)}^{2}x(\omega +\Delta \omega )x{\left(\omega +\Delta \omega \right)}^{H}]\\ & \;=\int_{D_{p}}{\left(\omega +\Delta \omega \right)}^{2}x(\omega +\Delta \omega )x{\left(\omega +\Delta \omega \right)}^{H}p_{a}(\Delta \omega )\;d\Delta \omega \\ & \;=\underset{k=1,\ldots ,N}{\Sigma }\;\underset{j=1,\ldots ,N}{\Sigma }\{\phi_{j}\phi_{k}^{H}({\psi_{j}}^{T}f){\left({\psi_{k}}^{T}f\right)}^{*}\\ & \;\times [1-\omega_{j}S(\omega ,a(\omega ),\omega_{j})-\omega_{k}^{*}S(\omega ,a(\omega ),\omega_{k}^{*})+\omega_{j}\omega_{k}^{*}Q(\omega ,a(\omega ),\omega_{j},\omega_{k})]\}. \end{array}$$

This modal expression of the second moment can also be expressed in matrix form, by using expression (3.10) of the averaging matrix ${\hat{H}}^{\left(l\right)}$ and the general discussion of §3*a*. Basic algebra, together with the substitutions ${\hat{x}}^{\left(l\right)}(\omega )={\hat{H}}^{\left(l\right)}(\omega )f^{\left(l\right)}$, $\mathrm{var}[x^{\left(l\right)}(\omega )]=E[x^{\left(l\right)}(\omega +\Delta \omega )x^{\left(l\right)}{\left(\omega +\Delta \omega \right)}^{H}]-{\hat{x}}^{\left(l\right)}(\omega ){\hat{x}}^{\left(l\right)}{\left(\omega \right)}^{H}$, **P**_x_(**A**^(*l*)^_1_)^−1^**f**^(*l*)^=0 and $P_{x}{\left(A_{1}^{\left(l\right)}\right)}^{-1}A_{0}^{\left(l\right)}={\left[0-iI\right]}^{T}$, gives indeed

6.16 $$E[{\left(\omega +\Delta \omega \right)}^{2}x(\omega +\Delta \omega )x{\left(\omega +\Delta \omega \right)}^{H}]=\hat{d}(\omega )\hat{d}{\left(\omega \right)}^{H}+\mathrm{var}[d(\omega )],$$

where **d**(*ω*)=i*ω***x**(*ω*), $\hat{d}(\omega )$ is its frequency average, retrievable from the second part of the vector ${\hat{x}}^{\left(l\right)}(\omega )$ and *var*[**d**(*ω*)]=[**0** −**I**]*var*[**x**^(*l*)^(*ω*)][**0** −**I**]^T^.

In the typical particular case of the previous section, i.e. **A**(*ω*)=(**K**+*iω***C**−*ω*^2^**M**), the integrals of the kinetic energy, $T=\int_{t=-\infty }^{\infty }(1/2)(\partial x(t)/{\partial t}^{T})M(\partial x(t)/\partial t)\;dt$, can thus also be expressed in terms of computable averaged frequency densities thanks to these expressions.

*Full system energy*. Through theorem 6.3, one knows that the kinetic energy of the whole system is

6.17 $$\begin{array}{r} T=\frac{1}{4\pi }\underset{k=1,\ldots ,N}{\Sigma }\;\underset{j=1,\ldots ,N}{\Sigma }\left[m_{kj}\left(\int_{-\infty }^{\infty }\omega^{2}x_{j}(\omega )x_{k}{\left(\omega \right)}^{*}\;d\omega \right)\right] \end{array}$$

6.18 $$\begin{array}{r} =\frac{1}{4\pi }\underset{k=1,\ldots ,N}{\Sigma }\;\underset{j=1,\ldots ,N}{\Sigma }\left[m_{kj}\left(\int_{-\infty }^{\infty }E[{\left(\omega +\Delta \omega \right)}^{2}x_{j}(\omega +\Delta \omega )x_{k}{\left(\omega +\Delta \omega \right)}^{*}]\;d\omega \right)\right]=\hat{T}. \end{array}$$

This can further be expanded in terms of the average and variance of the response, by using equation (6.16), and it can finally be written in a compact matrix form similar to that of equation (6.9), i.e.

6.19 $$T=\hat{T}=\frac{1}{4\pi }\left\{\mathrm{tr}\left[M\int_{-\infty }^{\infty }\mathrm{var}[d(\omega )]\;d\omega \right]+\int_{-\infty }^{\infty }\hat{d}{\left(\omega \right)}^{H}M\hat{d}(\omega )\;d\omega \right\}.$$

*Component energy.* The kinetic energy of two individual components distinguished by indices ‘A’ and ‘B’, generally such that

6.20 $$d(\omega )=[V_{A}\;V_{B}]\left[\begin{array}{c} d_{A}(\omega )\\ d_{B}(\omega ) \end{array}\right]\;\mathrm{and}\;\left[\begin{array}{c} d_{A}(\omega )\\ d_{B}(\omega ) \end{array}\right]={\left[W_{A}\;W_{B}\right]}^{T}\;d(\omega )$$

is then

6.21 $$T_{j}=\frac{1}{4\pi }\left\{\mathrm{tr}\left[M_{jj}\int_{-\infty }^{\infty }\mathrm{var}[d_{j}(\omega )]\;d\omega \right]+\int_{-\infty }^{\infty }{\hat{d}}_{j}{\left(\omega \right)}^{H}M_{jj}{\hat{d}}_{j}(\omega )\;d\omega \right\},$$

where var[**d**_*j*_(*ω*)]=[**W**_*j*_]^T^var[**d**(*ω*)][**W**_*j*_]*, ${\hat{d}}_{j}(\omega )={\left[W_{j}\right]}^{T}\hat{d}(\omega )$ and $M_{jj}=V_{j}^{H}MV_{j}$, and *j*=A or B. Similarly, the integrated kinetic energy of the coupling between the two components is equal to *T*_AB_+*T*_BA_ where

6.22 $$T_{jk}=\frac{1}{4\pi }\left\{\mathrm{tr}\left[M_{jk}\int_{-\infty }^{\infty }\mathrm{cov}[d_{k}(\omega ),d_{j}(\omega )]\;d\omega \right]+\int_{-\infty }^{\infty }{\hat{d}}_{j}{\left(\omega \right)}^{H}M_{jk}{\hat{d}}_{k}(\omega )\;d\omega \right\}$$

for *j*,*k*=A or B.

The kinetic energy of a component made of a single degree of freedom, say the *j*th one such that **V**_A_=**e**_*j*_, is thus the following sum of two terms:

6.23 $$T_{A}=\frac{1}{4\pi }\left\{m_{jj}\left(\int_{-\infty }^{\infty }\mathrm{var}[d_{j}(\omega )]\;d\omega +\int_{-\infty }^{\infty }|{\hat{d}}_{j}(\omega ){|}^{2}\;d\omega \right)\right\}=\frac{m_{jj}}{4\pi }\int_{-\infty }^{\infty }E|d_{j}(\omega ){|}^{2}\;d\omega$$

where all the averages of the scalar response *d*_*j*_(*ω*) may be evaluated as discussed.

*Real Gaussian case.* As for the potential energy discussed in §6*b*, in the case of a real Gaussian average, the modal expressions only necessitate the functions *S*^(g)^(*ω*,*a*,*ω*_*j*_) and *Q*^(g)^(*ω*,*a*,*ω*_*j*_,*ω*_*k*_) and, as discussed in §8, the matrix expression of the average can be evaluated without requiring any modal analysis.

### (d) Retrieval of input, dissipated and other energy terms

Other energy terms can be similarly evaluated exactly by integrating frequency averaged quantities. For example, the energy dissipated by viscous damping necessitates the evaluation of a term $(1/2\pi )\int_{-\infty }^{\infty }\omega x(\omega )x{\left(\omega \right)}^{H}\;d\omega =(1/2\pi )\int_{-\infty }^{\infty }E[(\omega +\Delta \omega )x(\omega +\Delta \omega )x{\left(\omega +\Delta \omega \right)}^{H}]\;d\omega$ whose second expression can be evaluated using equation (6.14).

The integral of the transient input power can also be evaluated in terms of the averaged expressions. For example, in the case of an impulse force, its frequency density is constant so that the total input energy is

6.24 $$E^{\left(\mathrm{in}\right)}=\int_{t=-\infty }^{\infty }(f\delta {\left(t)\right)}^{T}\underline{x}(t)\;dt=\frac{1}{2\pi }\int_{\omega =-\infty }^{\infty }f^{T}x(\omega )\;d\omega$$

and this term can again be expressed in terms of a frequency average

6.25 $$E^{\left(\mathrm{in}\right)}={\hat{E}}^{\left(\mathrm{in}\right)}=\frac{1}{2\pi }f^{T}\int_{\omega =-\infty }^{\infty }E[x(\omega +\Delta \omega )]\;d\omega =\frac{1}{2\pi }f^{T}\int_{\omega =-\infty }^{\infty }\hat{x}(\omega )\;d\omega .$$

### (e) Averaged square and averaged product of responses

The averaged square and product of responses did not appear in the expressions of the energy terms in the previous sections. They are however an important feature of the response at higher (mid or high) frequencies and are therefore an integral part of the computational goals of the proposed framework. Without these notions, a pure energy analysis indeed loses information on the phase of the transfer functions that may possibly be critical for some applications. Although these terms should not be overlooked, they may of course be omitted in situations where they are negligible, or in asymptotic forms, in the same way as the variance may be omitted in the asymptotic case of a *low-frequency*, deterministic, analysis.

## 7. Time domain analysis of the covariance and averaged products of responses

In this section, an interpretation of the second frequency moments is provided in the time domain. The discussion is similar to that of §5*a* where it was shown that, since frequency averaging with constant width is the convolution of an averaging function by the exact transfer function, its inverse transfer function is the product of the *exact* impulse response by the inverse Fourier transform of *p*_*a*_(Δ*ω*). Similar properties exist here with regards to *double* or *two-dimensional* inverse Fourier transforms of the second-order averaging terms, that is of the covariance and averaged products of responses. These properties are now detailed, with a particular focus on the real Gaussian case.

Starting from the definition of the inverse Fourier transform of equation (2.2), one finds

7.1 $$\begin{array}{rl} \mathrm{csq}[x_{A}(\omega_{A}),x_{B}(\omega_{B})] & =\int_{D_{p}}x_{A}(\omega_{A}+u)x_{B}{\left(\omega_{B}+u\right)}^{T}p_{a}(u)\;du\\ & =\int_{-\infty }^{\infty }{\underline{x}}_{A}(t_{A})\int_{-\infty }^{\infty }{\underline{x}}_{B}{\left(t_{B}\right)}^{T}\mathrm{csq}[e^{-i\omega_{A}t_{A}},\;e^{-i\omega_{B}t_{B}}]\;dt_{B}\;dt_{A}, \end{array}$$

where the integral in $\mathrm{csq}[e^{-i\omega_{A}t_{A}},\;e^{-i\omega_{B}t_{B}}]=e^{-i\omega_{A}t_{A}}\;e^{-i\omega_{B}t_{B}}\int_{D_{p}}p_{a}(u)\;e^{-iu(t_{A}+t_{B})}\;du$ needs to be evaluated for particular functions, *p*_*a*_(.). Following the same derivation, for the case of the power term, one finds

7.2 $$\begin{array}{rl} E[x_{A}(\omega_{A}+\Delta \omega ),x_{B}^{H}(\omega_{B}+\Delta \omega )] & =\int_{D_{p}}x_{A}(\omega_{A}+u)x_{B}{\left(\omega_{B}+u\right)}^{H}p_{a}(u)\;du\\ & =\int_{-\infty }^{\infty }{\underline{x}}_{A}(t_{A})\int_{-\infty }^{\infty }{\underline{x}}_{B}(t_{B})\mathrm{csq}[e^{-i\omega_{A}t_{A}},\;e^{+i\omega_{B}t_{B}}]\;dt_{B}\;dt_{A}. \end{array}$$

*Real Gaussian case.* In the real Gaussian case, *p*_*a*_(.)=*p*^(g)^_*a*_(.), one can again use equation (5.6), which gives

7.3 $$\int_{D_{p}}p_{a}^{\left(g\right)}(u)\;e^{-iu(t_{A}+t_{B})}\;du=e^{-{\left(t_{A}+t_{B}\right)}^{2}a^{2}/2}$$

and therefore

7.4 $$\begin{array}{rl} & {\mathrm{csq}}^{\left(g\right)}[x_{A}(\omega_{A}),x_{B}(\omega_{B})]\\ & \;=\int_{-\infty }^{\infty }\left(\int_{-\infty }^{\infty }\left\{{\underline{x}}_{A}(t_{A}){\underline{x}}_{B}{\left(t_{B}\right)}^{T}e^{-[{\left(t_{A}+t_{B}\right)}^{2}a^{2}/2]}\right\}e^{-i\omega_{B}t_{B}}\;dt_{B}\right)e^{-i\omega_{A}t_{A}}\;dt_{A} \end{array}$$

and

7.5 $$\begin{array}{r} E[x_{A}(\omega_{A}+\Delta \omega ),x_{B}{\left(\omega_{B}+\Delta \omega \right)}^{H}] \end{array}$$

7.6 $$\begin{array}{r} =\int_{-\infty }^{\infty }\left(\int_{-\infty }^{\infty }\left\{{\underline{x}}_{A}(t_{A}){\underline{x}}_{B}{\left(-t_{B}\right)}^{T}e^{-[{\left(t_{A}+t_{B}\right)}^{2}a^{2}/2]}\right\}e^{-i\omega_{B}t_{B}}\;dt_{B}\right)e^{-i\omega_{A}t_{A}}\;dt_{A}, \end{array}$$

where, in the last expression, the argument of ${\underline{x}}_{B}$ is negative. The variance derives directly from this expression.

**Figure 7.**
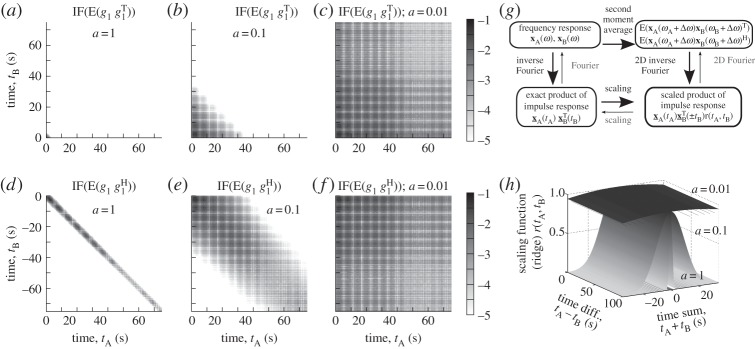
Illustration of the two-dimensional inverse Fourier transform of the second frequency moments as described in (*g*). Three averaging width, *a*=1, 0.1 and 0.01 rad Hz, are considered as well as two kinds of second frequency moment terms: plots (*a*), (*b*) and (*c*) are the maps of non-zero values of the inverse Fourier transforms of the frequency averaged products, *csq*^(g)^[*g*_1_(*ω*_A_),*g*_1_(*ω*_B_)], whereas plots (*d*), (*e*) and (*f*) are those of the power term E[*g*_1_(*ω*_A_+Δ*ω*),*g*_1_(*ω*_B_+ Δ*ω*)^H^], both for a real Gaussian average. They are respectively labelled ‘$\mathrm{IF}(E[g_{1}g_{1}^{T}])$’ and ‘$\mathrm{IF}(E[g_{1}g_{1}^{H}])$’. While the specific *g*_1_ transfer function of §4 is used here for illustration, the same Gaussian *ridge* scaling functions, *r*(*t*_A_,*t*_B_) presented in (*h*), would affect the product of the exact impulse responses of any general system similarly averaged.

Each of the two expressions (7.4) and (7.6) is a two-dimensional Fourier transform in the plane (*t*_A_,*t*_B_) of the product of an outer product of ${\underline{x}}_{A}$ and ${\underline{x}}_{B}$ by the weighting function, $r(t_{A},t_{B})=e^{-[{\left(t_{A}+t_{B}\right)}^{2}a^{2}/2]}$. While similar expressions would exist for other averaging distributions, the weighting function in the real Gaussian case, is remarkably also a Gaussian. Furthermore, since both impulse responses ${\underline{x}}_{A}$ and ${\underline{x}}_{B}$ are zero for negative value of their arguments, in the case of a causal system, and since ${\underline{x}}_{B}$ has a negative argument in the expression of the second average, these inverse Fourier transforms of the average functions result in functions that are each non-zero only in a single quadrant. In both cases, the outer product of the impulse responses is scaled by the Gaussian *ridge* function, *r*(*t*_A_,*t*_B_), that becomes tighter and tighter for increasing values of *a*. The maps of the non-zero components of these functions are illustrated in figure 7 for the transfer function *g*_1_, which was introduced in §4.

Different behaviours are visible for different averaging widths. For the smallest, *a*=0.01, the averaged products and covariance are very close to the non-averaged outer products of impulse responses. Simplifications however occurred since irrelevant details have been smoothed out of the response. This corresponds to a *low-frequency* behaviour. At the largest, *a*=1, the cross-influences are negligible while the energy information is preserved. This is the *high-frequency* behaviour. For the intermediate value, *a*=0.1, additional information about the coupling between responses at different times, i.e. phase in the frequency domain, is partially present. Observing both the covariance and averaged products provides a rational and quantitative tool with which to assess the actual behaviour of the dynamic system. As such, the quality of the existing methods to deal with the frequency methods can be analysed and compared by evaluating how well they preserve the actual frequency average, covariance and averaged product characteristics of the actual system. New methods can also be developed based on the actual important system features identified by the averaging process.

## 8. Efficient evaluation

Besides the asymptotic approximations, which provide evaluation of the averages that are accurate and economic in particular situations, the question of evaluating the averages efficiently and precisely in a general context is a relevant concern. As mentioned in §3*e*, a solution has been offered in [31] for the first moment averages, through the precise expansion of distribution or weighting functions, *p*_*a*_(Δ*Ω*), into fast converging rational expressions. Consequently, the exact averages are approximated in terms of fast converging rational functions of the poles of the system. These rational functions may be expressed in matrix form and evaluated exactly through stable Krylov projection methods, using only solutions of a system with added damping. Combining the projection subspaces at only a few interpolation frequencies can then provide a precise evaluation of the average function over a whole frequency range. This approach has been shown to be extremely efficient and able to provide the Gaussian averaged response in a whole frequency range, and with frequency varying averaging width, with only a few responses—as little as two—of the full system. This has particularly proved valuable to obtain the response of the system through *a single reduced model* of the system that gives different *low- to high-frequency focus* through different frequency ranges. The same strategy of mixing rational expressions, interpolation, and model reduction is applicable to other weighting functions that may or may not be originally expressed in rational form. In terms of asymptotic approximations, different approaches can be used, for example with rational expansions being used at zero, intermediate or infinite frequency.

The question of the efficient approximation or representation of the covariance and averaged product is of essential importance for the fundamental understanding of the *mid-frequency* region. The framework and analysis of this paper offers a new point of view, notably in that the pairs of plots (*a*)–(*d*), (*b*)–(*e*), (*c*)–( *f*) in figure 7 expose that the relevant patterns are the product of an outer product of functions in the (*t*_A_,*t*_B_) plane and another outer product of a Gaussian with a constant functions in the (*t*_A_+*t*_B_,*t*_A_−*t*_B_) plane. The consideration of the averaged product of responses additionally to the covariance is important in assuring this representation, as well as in preserving information on the phase, as previously mentioned. It is evident from the analysis and plots that it can, in theory, be retrieved from the covariance or energy information evaluated with a single averaging width over the whole frequency range.

The question of optimality of the approximations of the responses as in [19] can be reframed and possibly extended in the proposed averaging framework.

## 9. Conclusion

A solution framework for dynamic systems has been proposed that targets directly the frequency average of their responses and their higher, second frequency moments. This approach is applicable exactly, independently of the system's modal density or modal sensitivity so that a smooth transition from one region of the frequency spectrum to the other can be achieved by merely tuning an averaging width function, *a*(*ω*). This parameter can be interpreted as the typical standard deviation or the characteristic bandwidth of a distribution or weighting function *p*_*a*_(Δ*ω*). It describes the desired level of frequency precision or uncertainty of the solution at any frequency. Asymptotically zero values of the parameter correspond to a deterministic or *low-frequency* solution with zero variance, while larger values correspond to *high-frequency* solutions that can be understood in a more energetic or statistical sense. The transitional *mid-frequency* region is smoothly supported and no distinction or boundaries between the regions is necessary. The frequency averaged power or energy of the system, which are statistics that are usually considered at high-frequency, are found as function of the frequency average and of the averaged square of the absolute value of the response. It has been explicitly stressed that this fact considered in [38] for the case of a uniform distribution can be applied for a general weighting function. The general explicit analytical expressions are provided and demonstrated here for the case of a real Gaussian averaging function. Again, the flexibility in the choice of the frequency-dependent parameter, *a*(*ω*), allows to extract deterministic, statistical or intermediate frequency averaged power, with values of the parameter that are respectively small, large or intermediate. Contrary to the case of many statistical energy analysis and high-frequency methods, the power or power average is not the only result of the analysis in the proposed framework. The consideration of both frequency average and variance, and of the tunable uncertainty parameter, *a*(*ω*), in a single analysis gives a smooth transition from low- to mid- and high-frequency ranges and provides a framework in which the usually distinct *deterministic* and *statistical* approaches can be seen just as particular or asymptotic cases.

Many existing low-, mid- and high-frequency approaches can actually be placed and examined in the proposed framework. For example, deterministic modes, models of low-frequency physical components, such as springs and masses, SEA systems, analytical waves or hybrid models can be integrated into a single model. The coupling between any of these components within the framework must be characterized in such a way that the average, averaged square and variance of the responses of the global system are accurately represented. This may be seen as a generalization of the fact that the coupling of *high-frequency* or SEA components must be such that the transfer of energy in a whole SEA system is accurately represented and that the coupling between *low-frequency* or deterministic systems must be such that the deterministic transfer functions, i.e. their frequency average for zero value of the *a*(.) parameter, of the coupled system are properly predicted. Coupling conditions assuring matching of the averages and variance also offer a point of view for the coupling conditions between the deterministic and SEA components of hybrid models [46,47]: for the same averaging width, *a*(*ω*), a sub-system might behave in its low-frequency regime while another behaves in its high-frequency regime in which case, this would be reflected in the average and variance of the response of the components. Low-, mid- and hybrid mid-frequency coupling conditions are also particular cases of the more general framework coupling conditions. They are perfectly valid within the framework but only under certain conditions and for specific ranges of values of the parameter *a*(.). This is exactly in the same manner as the validity of a simplified model of a spring or a mass starts breaking down when the frequency increases and the wavelengths become closer to the dimensions of the actual physical spring or mass. Other topics of the literature that have not yet been covered here but would be worth studying in future works include the averaging in the location of force, measurement and interface areas, the treatment of higher moment such as the variance of the general energy terms, and the conjunction of frequency and statistical averages. On the other hand, material more rarely covered in the literature, that has been studied and highlighted here includes the treatment of the averaged product of responses and the covariance terms and the interpretations of the averages in the time domain. An efficient approach based on the frequency averages to estimate time responses has notably been proposed.

Besides the fact that many approximate or asymptotic methods can be integrated into a single analysis, the framework also provides the advantage of offering a natural environment in which they can be better understood and expanded. The precision of various methods to approximate low-, mid- or high-frequency components can also be studied in reference to how they affect the precision of the average and variance of transfer functions.

## Footnotes

1 Note that the reduced damping matrix was defined as *η* times the reduced stiffness matrix.
